# Neuro-perceptive discrimination on fabric tactile stimulation by Electroencephalographic (EEG) spectra

**DOI:** 10.1371/journal.pone.0241378

**Published:** 2020-10-28

**Authors:** Jiao Jiao, Xiaoling Hu, Yanhuan Huang, Junyan Hu, Chihchia Hsing, Zhangqi Lai, Calvin Wong, John H. Xin

**Affiliations:** 1 Institute of Textiles and Clothing, The Hong Kong Polytechnic University, Hong Kong, China; 2 Department of Biomedical Engineering, The Hong Kong Polytechnic University, Hong Kong, China; Georgia State University, UNITED STATES

## Abstract

The precise evaluation of sensory perceptions during fabric-skin interactions is still poorly understood in neuroscience. This study aims to investigate the cortical sensory response to fabric stimuli with different textiles by Electroencephalographic (EEG) spectral intensities, and evaluate the relationships between EEG frequency bands, traditional subjective questionnaires, and the materials’ physical properties. Twelve healthy adult participants were recruited to test three fabrics with different textile compositions of 1) cotton, 2) nylon, and 3) polyester and wool. The physical properties of the fabrics were quantitatively evaluated by a Fabric Touch Tester (FTT). Subjects were invited to rate the sensory perception of the fabric samples via a subjective questionnaire and objective EEG recording. Significant differences in the EEG relative spectral power of Theta and Gamma bands were acquired in response to the different fabric stimuli (*P*<0.05). The Theta and Gamma powers demonstrated a significant correlation with the most of the subjective sensations evaluated by questionnaire and the fabrics’ physical properties by FTT (*P*<0.05). The EEG spectral analysis could feasibly be used for the discrimination of fabric stimuli with different textile compositions and further indicates sensory perceptions during fabric stimulation. This finding may provide evidence for further exploratory research of sensory perceptions via EEG spectral analysis, which could be applied to the study of brain generators of skin tactility in future prostheses and the automatic detection of sensory perception in industries.

## Introduction

Tactile sensation is considered somatic, and refers to the sense of touch originating at the body’s surface, transmitted via peripheral nerves to the central nervous system and neural computation for recognition. The investigation of tactile sensation involves research of human physiology, psychology, and biomedical engineering, which can be widely applied in the area of rehabilitation, the innovation of electronic skin and prostheses, and quality evaluation of textile comfort. Numerous and precise sets of sensory data feedback are required to address the gaps of signal processing algorithms during prosthetic control and stroke patients’ sensory deficits when detecting motion intention. In order to collect the sensory perceptions of human subjects, the sensory evaluation of various fabrics, e.g., textile fabrics, via rated questionnaires has been applied during a selection of studies [1, 2]. However, the results obtained from questionnaires may vary among individuals due to a number of disturbances related to cognition, physiology, psychology, or even social background [3, 4]. Moreover, individuals with neurological impairments in communication, sensorimotor function, and/or cognition, such as post-stroke patients, may find it difficult to precisely express their feelings or perceptions via such a method [1, 2]. However, objective and quantitative measures on sensory perceptions are still lacking, because of the limited understanding on the sensory responses in the brain.

In the textile industry, instruments have been developed for quantitative measurement on physical properties of different textile fabrics, such as friction, stiffness and roughness, with the understanding that these properties directly affect the human tactile sensation and inform the perceived comfort of clothing [1, 5]. For example, the instrument of Fabric Touch Tester (FTT), which allows a comprehensive evaluation of the aspects of fabric thermal effects, compressive capability, mechanical bending, fabric shearing, and surface friction using a single sample [6]. Researchers have attempted to correlate the physical outputs as measured by FTT with the subjective fabric sensation rated by questionnaires, attempting to predict the direct relationship between the fabric’s physical properties and the sensory perception after loading the material [1, 6, 7]. While an urgent need is to find an objective and quantitative relationship between the neurological sensation and the fabric’s physical properties, with the purpose to minimize the subjective biases.

Quantitative and objective methods are required for investigating the correlation between the physical properties of a fabric and sensory perception in response to a fabric stimulus in the nervous system. However, the methods outlined in the literature are still preliminary. Non-invasive methods, like functional magnetic resonance imaging (fMRI) and electroencephalography (EEG), were adopted for direct capturing of the neural responses in the nervous system [2, 8, 9]. For example, fMRI has been utilized to identify the neural pathways related to the fabric stimuli at different sites of the forearm, during which 13 healthy participants were recruited to test four fabrics using a robotic caressing device [9]. However, fMRI is not ideal to reveal the transient neural responses to tactile stimuli, because the temporal resolution of current fMRI is around 2s to 5s [10, 11]. Due to the sensory adaptation, the transient sensory neural responses will attenuate quickly during tonic tactile stimulation (in milliseconds) [10, 11]. Hoefer et al. [8] employed the event-related potential (ERP) in the time domain captured by EEG during fabric stimulation on the ventral side of the forearm to discriminate different fabric samples (n = 3). They observed non-coincidently different ERP amplitudes in 24 test subjects by varying fabric stimuli on the forearm. Nevertheless, the differences in such an amplitude of ERP in the time domain were not significant enough for the differentiation of fabric samples when applied to the forearm or the palm. This was because the ERP waveform analysis in the time domain required a high repetition (e.g., N>100) for the ensemble mean with a needed signal-to-noise ratio, which limited its resolution in the discrimination on different fabric stimuli with less repeated stimuli (e.g., N<5) in the experimental practice [12, 13]. EEG spectral analysis in the frequency domain is another alternative method in the investigation of neural activates [14], which has greater potential for industrial applications due to calculation simplicity, compared with EEG ERP in the time domain. Furthermore, EEG spectra in different frequency bands are more closely related to the brain’s sensorimotor status than the overall ERP amplitudes in the time. For instance, energy changes in the Alpha (8 Hz-13 Hz) and Beta (13 Hz-30 Hz) bands of the EEG signals have been employed in real-time motor imagery identification for rehabilitation applications, e.g., brain-computer interfacing systems for disabilities [15]. The Alpha wave collected from six healthy subjects was found to be positively correlated with the applied pressure from sample shirts during static wearing [2]. An increase of Gamma (30 Hz-100 Hz) is usually associated with high-level cognitive activities like decision-making [16] and processing multiple sensory inputs [15, 17] ie. audio-visual synchronous stimuli [18]. The increase of EEG low-frequency components, e.g., Delta (0.1Hz-4Hz) and Theta (4Hz-8Hz) bands, could be related to respective sleeping and idling mental status, such as drowsiness, in adults [15, 19]. It is possible that EEG spectral intensities in the frequency bands could be the relatively sensitive measures for identifying different tactile stimuli. Although the EEG spectral analyses have been widely applied in various researches of neural activities, it has not been adopted in measuring the cortical sensory responses to fine tactile stimulation.

Therefore, the purpose of this study was to investigate the cortical sensory responses to fabric stimuli with different textile fabrics by EEG spectral analysis. We hypothesized that EEG spectral intensities in different bands could discriminate the fabric stimuli with different textile compositions. Relationships could be found between EEG frequency bands, traditional subjective questionnaires, and the materials’ physical properties.

## Materials and methods

Three different fabric samples were selected with the main textile elements of 1) cotton, 2) nylon, and 3) a combination of polyester and wool. All three testing fabrics were prepared into 20cm×10cm pieces for investigation. The physical properties of the fabric samples were first quantitatively evaluated using FTT. Human participants were then invited to rate the sensory perception on the samples via a subjective questionnaire and by objective EEG recording during static loading of the fabric samples on the forearm.

### Physical evaluation of fabric by FTT

The detailed specifications of the selected fabric samples are shown in Table 1. These are widely used textiles. FTT (SDL Atlas, U.S.) was used to measure the physical properties in the aspects of fabric thermal characteristics, surface, bending, and compression with detailed sub-parameters [6] (as shown in Table 2). Each type of fabric was tested three times via FTT. Among them, the mean value obtained from the warp and weft directions was treated as one experimental reading for the parameters of bending average rigidity (BAR), bending work (BW), surface friction coefficient (SFC), surface roughness amplitude (SRA), and surface roughness wavelength (SRW). Fabric A (100% cotton with plain weave) was chosen as the control, since it is commonly used in the clothing industry and widely perceived as one of the most comfortable and acceptable fabrics. Fabric B was composed of 87% nylon mixed with 13% elastane, which could result in a cool feeling when applied, due to its relatively high thermal conductivity. Fabric C consisted of 60% polyester and 40% wool, which could provide a feeling of warmth, while being much lighter in weight and smoother in terms of surface than fabric comprising 100% wool.

**Table 1:** Textile composition of the three sample fabrics.

Fabric no.	Fabric Description	Component	Weight (g/m^2^)	Thickness (mm)
A	100% Cotton	Plain Woven	127.7±0.8	0.39±0.01
B	87% Nylon/ 13% Elasthan	Jacquard	113.3±1.3	0.77±0.00
C	60% Polytester/ 40% Wool	Flannel Woven	340.8±2.4	1.29±0.01

**Table 2:** Physical parameters measured by FTT.

Modules	Description	Indices	Unit
Compression	Compression Work	CW	gf*mm
	Compression Recovery Rate	CRR	Non-unit
	Compression Average Rigidity	CAR	gf*mm^-3^
	Recovery Average Rigidity	RAR	gf*mm^-3^
Thermal	Thermal Conductivity when Compression	TCC	W*m^-1^*K^−1^
	Thermal Conductivity when Recovery	TCR	W*m^-1^*K^−1^
	Maximum Thermal Flux	Qmax	W*m^-2^
Bending	Bending Average Rigidity	BAR	gf*mm*rad^-1^
	Bending Work	BW	gf*mm*rad
Surface	Surface Friction Coefficient	SFC	Non-unit
	Surface Roughness Amplitude	SRA	μm
	Surface Roughness Wavelength	SRW	mm

### Participants

After obtaining ethical approval from the Human Subjects Ethics Committee of The Hong Kong Polytechnic University (Approval number: HSEARS20150812002), 12 healthy adults (4 males, 8 females) were recruited to take part in this study. The demographic data of the subjects are as follows (mean ± standard deviation (SD)): age, 24.1 ± 3.4 years; height, 166.7 ± 6.5 cm; weight, 58.0 ± 8.9 kg; body mass index (BMI), 20.9 ± 3.2 kg·m^−2^. None of the participants had any history of neurological, psychiatric, and/or cardiovascular disease, and had enough cognition to follow simple instructions and understand the objectives of the study. Moreover, experimental process and possible risks were explained to each participant prior to data collection. Written informed consent form was obtained from each person for taking part in the study prior to their involvement.

### Sensory perception detected by EEG

**Fig 1:**
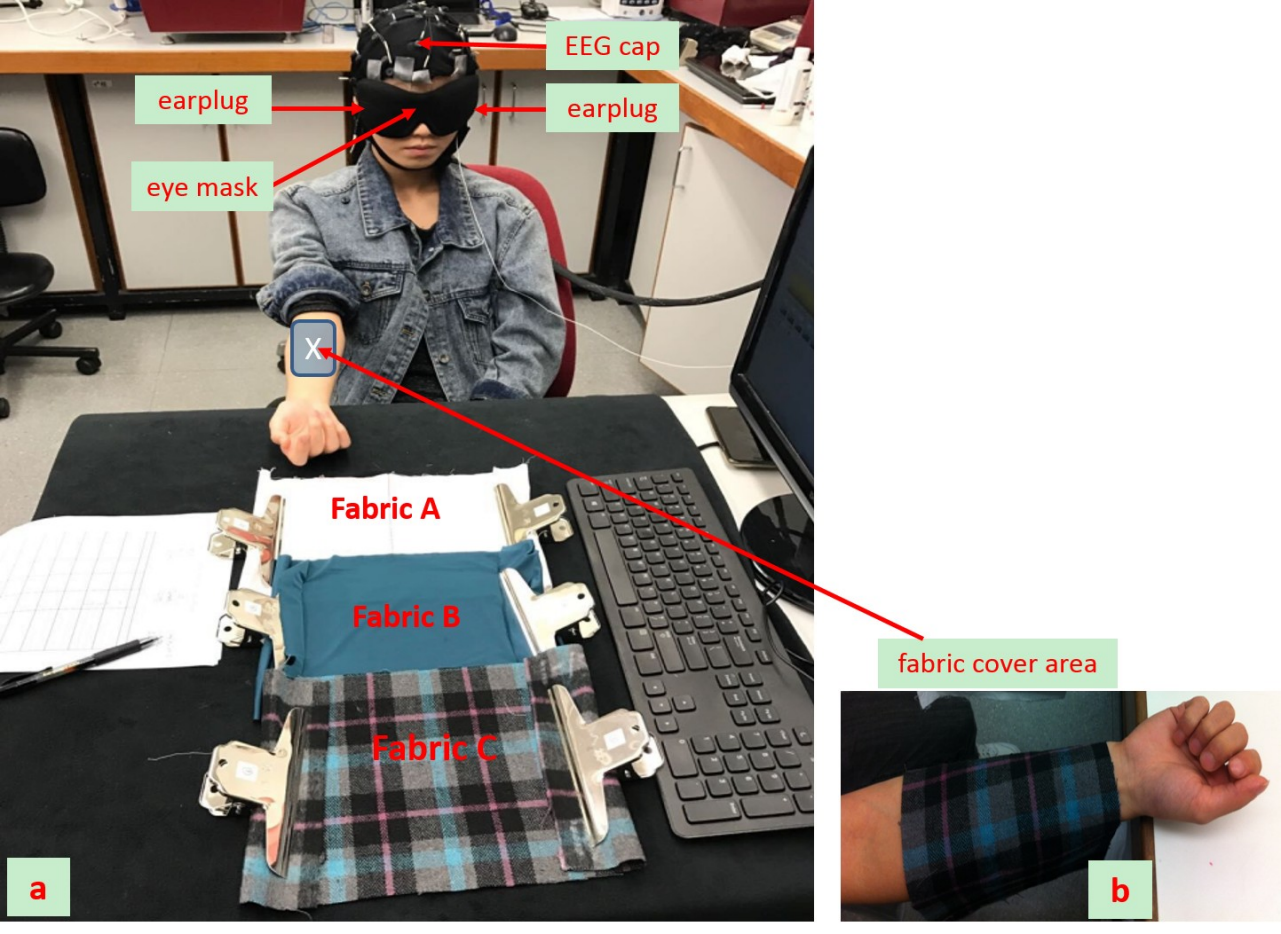
Experimental setup for the EEG evaluation during the fabric stimuli.

The experiment on human participants was conducted in a quiet room with a controlled environmental temperature of 24 ± 2°C and a relative humidity of 60% ± 5%. Each subject was first invited to conduct the objective measurement of the sensory perception test by EEG when fabric stimuli were applied. The experimental setup is provided in Fig 1A. The individual in this manuscript has given written informed consent (as outlined in PLOS consent form) to publish these case details. Participants were comfortably seated with the forearm of the dominant arm resting on a table. The dominant arm of all participants in this study was on the right side. During the test, visual and auditory stimuli from the environment were further minimized with the use of a blindfold and earplugs. An electroencephalography system with 64 channels (BP-01830, Brain Products Inc.) was mounted onto the scalp of each participant according to the standard 10–20 system, in order to record the whole brain EEG with the skin impedance of each channel below 5 KΩ [20].

**Fig 2:**
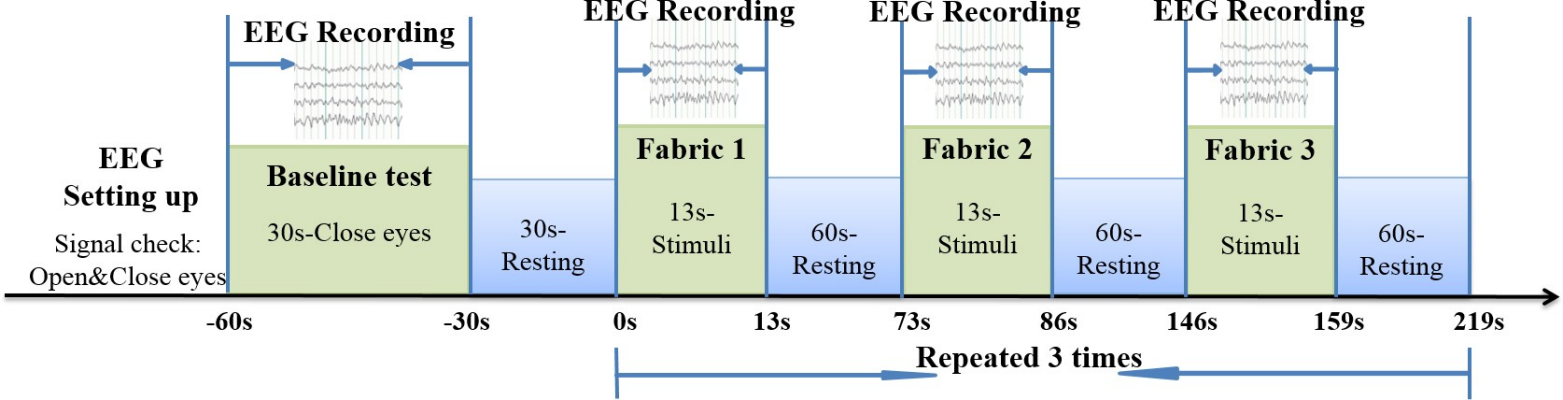
Experimental protocol for EEG evaluation.

The stimulation protocol, presented with the timeline, is summarized in Fig 2. The baseline detection in Fig 2 was a 30-second EEG recording taken when a subject closed their eyes and stayed awake, relaxed and calm. Following the baseline recording and in preparation of the subsequent EEG recording on fabric stimulation, during which no movement was allowed, the subject was permitted 30 seconds of activity (30s-Resting), such as slight body motions or eye blinking. There followed three episodes of tactile stimuli with the different fabrics, each of which was statically loaded (i.e., without striking) onto the skin surface of the ventral side of the forearm (Fig 1B) for 13 seconds. A 60-second resting interval took place between two consecutive fabric stimuli. During the fabric stimuli, participants closed their eyes while remaining awake without mental effort. The sequence of the stimuli for fabrics A, B, and C was randomized (fabrics 1, 2, and 3 in Fig 2 could be any one of fabrics A, B, or C, in an unplanned order). The cycle of three-fabric stimuli (from 0s to 219s) was repeated three times.

**Fig 3:**
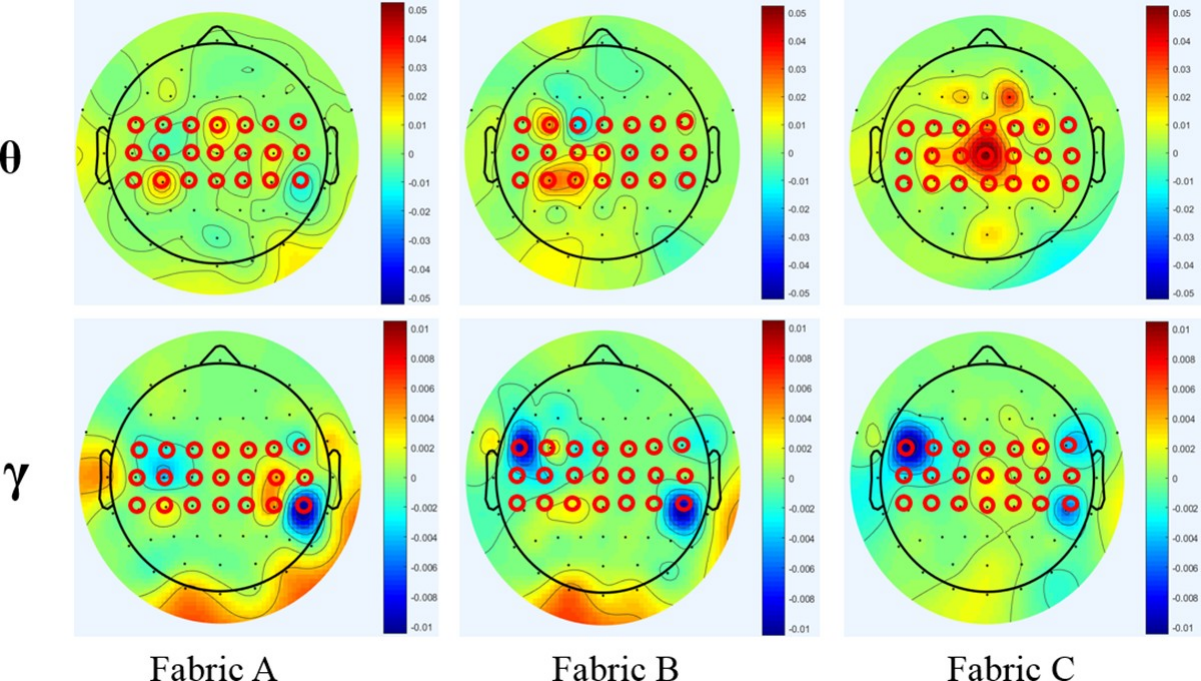
The whole brain EEG topography on the Theta and Gamma power bands in response to fabric stimuli. The topography about the relative spectral powers of 64 EEG channels on the whole brain indicated the power changes compared to the baseline. There is no unit for the relative power according to Eq 1, normalized with respect to the total power. Red markers indicate the 21 EEG channels selected (FC3, FC1, FC5, C5, C3, C1, CP3, CP1, CP5, FC2, FC4, FC6, C2, C4, C6, CP2, CP4, CP6, Cz, FCz, and CPz).

**Fig 4:**
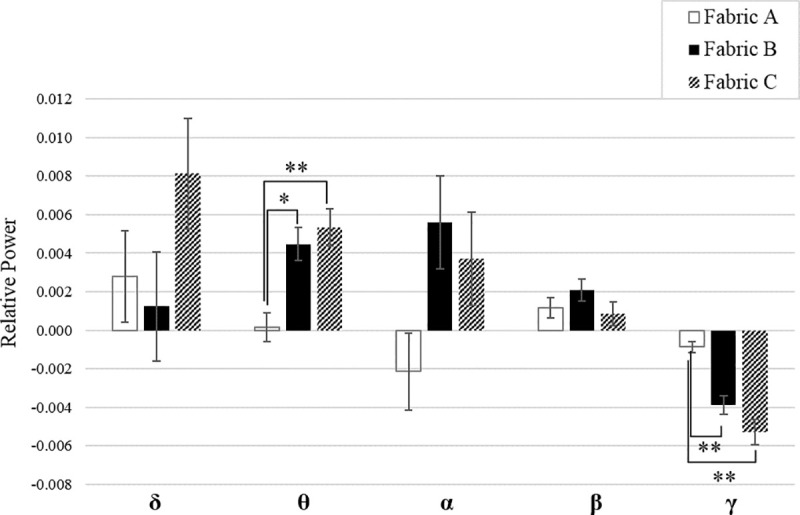
EEG relative power bands of 21 channels on the somatosensory area in response to fabric stimuli. The significant difference is indicated by **P <0.01 and * *P* <0.05 among three fabrics. Data are presented as mean ± SEM. Delta: δ; Theta: θ; Alpha: α; Beta: β; Gamma: γ. There is no unit for the relative power according to Eq 1, normalized with respect to the total power.

Real-time recording was performed using the EEG system, with a sampling frequency of 1000 Hz for offline processing. In this work, EEG signals were processed off-line by Matlab 2016 (MathWorks Inc.), after digitization with a sampling frequency of 1000 Hz. A 4^th^ order Butterworth band-pass filter from 0.1Hz -100 Hz without phase shift was applied to the EEG, to remove the DC offset and unrelated high frequency components, followed by a 4^th^ order Butterworth band-stop filter (without phase shift) from 49 Hz-51 Hz to remove the 50Hz noise from the environment. The Gamma oscillations in response to sensory stimulation would mainly situate in the lower Gamma band [21], i.e., <45Hz. The band-stop filtering affected little on the gamma band evaluation. Visual inspections on the EEG trials after the filtering were performed to check the signal quality, e.g., identification of motion artifacts and unwanted electromyography (EMG). There was no motion artifacts, or EMG, found in the recorded trials. The EEG signals related to each event, i.e., a trial of baseline and fabric stimuli, were then divided into individual segments, and the relative power of each EEG channel on each EEG frequency band, i.e., Delta (δ), Theta (θ), Alpha (α), Beta (β) and Gamma (γ), was calculated according to the following equation,

$$P_{Relative\;band}=\frac{\int_{F_{1}}^{F_{2}}p(f)df}{\int_{0}^{100}p(f)df}-Mean\left[\frac{\int_{F_{1}}^{F_{2}}p_{baseline}(f)df}{\int_{0}^{100}p_{baseline}(f)df}\right],$$ (1)

where, P_Relative band_ is the relative spectral power of a frequency band; p(f) is the power spectral density of an EEG segment for a fabric stimulating event, estimated by Welch’s method [22]; F_1_ and F_2_ are the cut-off frequencies of an EEG frequency band, as described in the introduction; and p_baseline_(f) is the power spectral density of the EEG segments in baseline. As indicated in Fig 2, the stimuli for each fabric was repeated thrice. Following their calculation, the EEG frequency bands’ mean value of the three-repeated EEG relative spectral powers of each single channel, a total of 64, was applied to calculate the relative power changes as compared to the baseline, and plot the EEG topography for fabric stimulation (Fig 3). However, the mean values of the EEG relative power of 21 channels for the frequency bands were used (Fig 4) when comparing the neural response to each band for each fabric stimuli, and exploring the relationship between the questionnaire ratings and physical properties measured by FTT.

### Subjective evaluation by questionnaire

Following the EEG recording, the participants’ subjective sensations of the stimulation by the three fabrics were evaluated with a questionnaire designed in accordance with the American Association of Textile Chemists and Colorists (AATCC) Evaluation Procedure 5 [23]. It was utilized to evaluate the thermal and tactile sensations of a subject during fabric stimulation. The thermal sensation was further measured by two sub-properties, i.e., cool-warm and damp-dry. Tactile sensation was evaluated via 10 sub-properties i.e., itchy-non-itchy, scratchy-non-scratchy, prickly-non-prickly, rough-smooth, sticky-non-adhesive, stiff-pliable, thick-thin, hard-soft, inelastic-elastic, non-fullness-fullness and the overall indicator of uncomfortable-comfortable. For each sub-property, a numeric scale from 1 to 7 was applied in the evaluation [1]. In the subjective sensory test, a participant was seated with the same configuration as in the EEG test, with the testing forearm placed on the table (Fig 1B). The subjects still wore the blindfold, but did not wear earplugs. Each fabric was statically loaded onto the target skin surface as in the EEG test. Thereafter, the participants were asked each question in the questionnaire for rating.

### Statistical analysis

**Fig 5:**
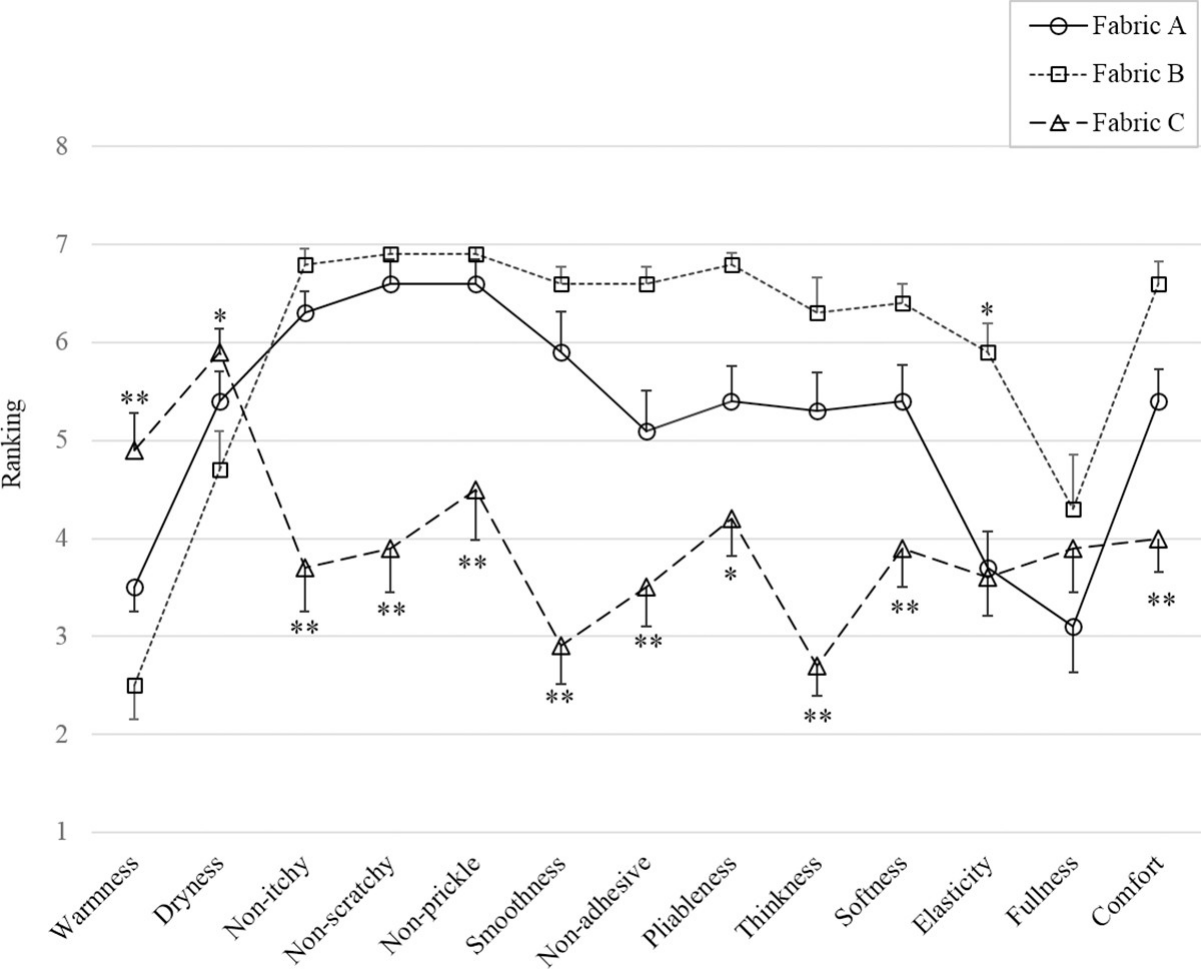
Rates of the subjective sensations measured by questionnaire in response to fabric stimuli. The significant difference is indicated by **:*P* <0.01 and *:*P* <0.05, as compared with the other two fabrics. Data are presented as mean ± SEM. Warmness: Cool-Warm; Dryness: Damp-Dry; Non-itchy: Itchy-Non-itchy, Non-scratchy: Scratchy-Non-scratchy; Non-prickly: Prickly-Non-prickly; Smoothness: Rough-Smooth; Non-adhesive: Sticky-Non-adhesive; Pliableness: Stiff-Pliable; Thickness: Thick-Thin; Softness: Hard-Soft; Elasticity: Inelastic-Elastic; Fullness: Non-fullness-Fullness; Comfort: Overall uncomfortable-Comfortable. The ranking scale is from 1 to 7 [1].

All analyses were conducted using SPSS package version 16.0 (SPSS Inc., USA). One-way analysis of variance (ANOVA) was carried out to investigate the subjective sensation parameters and the relative spectral powers of EEG frequency bands on the selected 21 channels with respect to the three fabric samples. When a significant difference was discovered, Bonferroni post hoc tests were performed to examine the pairwise comparison among the three fabrics (Fabric A vs B, Fabric A vs. C, and Fabric B vs. C, total thrice). The significance level of the Bonferroni post hoc test was set at 0.05, and the level of *P*<0.01 was also indicated in the related figures (Figs 4 and 5).

Bivariate correlation was applied to explore the relationship between the individual EEG band powers and subjective sensations measured by questionnaire (EEG v.s. Sub-sensation), and the correlation between the EEG band powers and the physical properties by FTT collected from the three textile fabrics (EEG v.s. Phy.). In the case of EEG v.s. Sub-sensation, a correlation pair was located by linking a reading of a channel’s EEG band power to a sensation reading in the questionnaire taken during the same fabric stimulation. There were 21 channels of EEG reading during a fabric stimulation, 3 textile fabrics and 12 subjects, resulting in a total of 756 pairs of data in the correlation on each sensation (Table 6). Similarly, there were 756 pairs of data for the EEG v.s. Phy. correlation (Table 7), although it was understood that different EEG readings could be related to the same value of a sensation or a fabric’s physical property due to the relatively low resolutions in the questionnaire and physical tests of FFT. Similar correlation analyses were adopted during textile stimulation of previous studies, such as Wang et al.’s [2] and Singh et al.’s [9] to investigate the relationship between electrophysiological signals (multi-channel EMG and EEG) versus subjective sensations (by questionnaire), as well as versus fabric physical properties.

## Results

### Fabric physical properties by FTT

Table 3 shows the fabric physical properties in the aspects of compression, thermal, bending, and surface measured by FTT. Fabric B had the smallest compression work (CW) (117.9gf*mm) among the three samples. CW describes the deformation of fabrics by simulating the change of thickness when force is applied to a sample [24]. Fabric C had the largest CW of 1339.0 gf*mm, while Fabric A had a value of 345.4 gf*mm. Qmax measures the maximum heat flow transferred through the fabrics when in contact with simulated skin. A high Qmax value could result in a subjective perception of coolness [7]. Fabric B had the highest Qmax (1745W*m^-2^) compared to the other two (Fabric A: 1467.8 W*m^-2^ and Fabric C: 633.1W*m^-2^). The bending parameters mainly refer to the rigidity of fabrics, and were believed to potentially reflect stiffness perceptions [7]. Fabric C demonstrated the highest bending rigidity mainly due to thickness, as measured by BAR and BW. The fabric surface smoothness was characterized by SFC, SRA, and SRW. SFC describes the overall friction, while SRA and SFC are related to the fabrics’ weaving specifications [7]. Fabric B had the lowest surface friction (SFC) and the smallest SRA, meaning it was the smoothest among the three.

**Table 3:** Physical properties of the three fabrics obtained from FTT.

Fabric Inner	Compression				Thermal			Bending			Surface		
	CW	CRR	CAR	RAR	TCC	TCR	Q_max_	BAR		BW	SFC	SRA	SRW
A	345.4±12.7	0.46± 0.02	7.9± 0.4	18.3±0.6	0.05± 0.00	0.05± 0.00	1467.8±18.6	188.1± 7.2	561.7±11.1		0.23±0.01	16.7±2.0	1.9± 0.1
B	117.9±7.0	0.59± 0.03	28.4±1.7	53.4±4.0	0.05± 0.00	0.05± 0.00	1745.0±29.3	152.4± 10.2	219.5±14.2		0.16±0.01	8.3± 1.1	1.9± 0.2
C	1339.0±33.6	0.65± 0.00	1.9± 0.0	2.8± 0.1	0.06± 0.00	0.06± 0.00	633.1± 3.1	349.5± 9.6	1160.5±11.6		0.29±1.01	40.0±3.3	3.0± 0.3

CW: Compression Work(gf*mm); CRR: Compression Recovery Rate (Non-unit); CAR: Compression Average Rigidity(gf*mm^-3^); RAR: Recovery Average Rigidity(gf*mm^-3^); TCC: Thermal Conductivity when Compression(W*m^-1^*K^−1^); Qmax: Maximum Thermal Flux(W*m^-2^); TCR: Thermal Conductivity when Recovery(W*m^-1^*K^−1^); BAR: Bending Average Rigidity(gf*mm*rad^-1^); BW: Bending Work(gf*mm*rad); SFC: Surface Friction Coefficient (Non-unit); SRA: Surface Roughness Amplitude(μm); SRW: Surface Roughness Wavelength (mm).

### Sensory perception detected by EEG

Fig 3 is EEG topography shows EEG relative power changes in the Theta and Gamma bands of 64 channels on the whole brain as compared to the baseline. Stimulation of fabrics B and C achieved strong responses in both bands. For Theta band, the hot spots related to the significant relative power changes captured were mainly located in the contralateral and central areas. However, the hot spots in the Gamma band were discovered mainly in the bilateral areas.

In Fig 3, 21 EEG channels covering the primary somatosensory area of the cerebral cortex were selected and marked in red, including FC3, FC1, FC5, C5, C3, C1, CP3, CP1, CP5, FC2, FC4, FC6, C2, C4, C6, CP2, CP4, CP6, Cz, FCz, and CPz. Data taken from the 21 EEG channels of the three-fabric stimuli was used for further investigation, eg. ANOVA analysis among the three-fabric stimuli and correlation.

Fig 4 demonstrates the differentiation on the three fabric samples by EEG relative power in the frequency bands collected from the 21 EEG channels over the primary somatosensory area. Significant power differences were captured in the Theta (F = 7.2, *P* = 0.001) and Gamma bands (F = 10.2, *P =* 0.000) by the analysis of the one-way ANOVA. The Bonferroni post hoc test was subsequently administered to obtain a pairwise comparison between each two fabrics. The Theta band’s relative power in response to the stimulation of Fabric C was significantly higher than those of Fabric A (post hoc tests, *P* = 0.001), and Fabric B (post hoc test, *P* = 0.008). Fabric stimulation reduced the relative Gamma power compared with the baseline state, and resulted in negative relative power values. The reduction in the Gamma power in response to Fabric C was larger than that of Fabric A (post hoc tests, *P* = 0.001), and Fabric B (post hoc test, *P* = 0.01). However, no significant difference was found in other frequency bands. The detailed statistical results, including the effect on differentiation by EEG relative powers, are listed in Table 4.

**Table 4:** EEG relative power bands of 21 channels on the somatosensory area in response to fabric stimuli.

Band	Mean (95% Confidence Interval)			One-way ANOVA	
	Fabric A	Fabric B	Fabric C	*P* (Partial η^2^)	*F*
**Delta**	2.8E-03	1.2E-03	8.1E-03	0.33 (0.003)	1.11
	(-4.0E-03 to 10E-03)	(-5.0E-03 to 8E-03)	(1.0E-03 to 15E-03)		
**Theta**	0.1E-03	1.5E-03	5.3E-03	0.001** (0.021)	7.17
	(-2E-03 to 2E-03)	(2E-03 to 6E-03)	(3E-03 to 7E-03)		
**Alpha**	-2.1E-03	5.6E-03	3.7E-03	0.166 (0.005)	1.80
	(-8E-03 to 4E-03)	(0E-03 to 11E-03)	(-2E-03 to 10E-03)		
**Beta**	1.2E-03	2.1E-03	0.8E-03	0.528 (0.002)	0.64
	(0 E-03 to 3E-03)	(1E-03 to 4E-03)	(-1E-03 to 2E-03)		
**Gamma**	-0.9E-03	-3.0E-03	-5.3E-03	0.000** (0.03)	10.24
	(-2E-03 to 1E-03)	(-5E-03 to -2E-03)	(-7E-03 to -4E-03)		

**P <0.01

* *P* <0.05; There is no unit for the relative power according to Eq 1, normalized with respect to the total power.

### Subjective evaluation by questionnaire

The results of the subjective sensory rating of the three fabric samples collected via questionnaire are depicted in Fig 5 and Table 5. Significant subjective sensory differences were identified in all properties of the three different fabrics (One-way ANOVA, *P*<0.05), with the sole exception of fullness. The Bonferroni post hoc test was conducted on each subjective sensory item to acquire a pairwise comparison between the three fabrics. The subjective sensory rating of Fabric C indicated significant differences in many properties (post hoc test, *P*<0.05), except Elasticity and Fullness, compared to the other two. The subjective sensory rating only highlighted a significant difference on the property of Elastic between Fabrics A and B (post hoc test, *P* = 0.000), and Fabrics B and C (post hoc test, *P* = 0.000), with no significant difference between Fabrics A and C (post hoc test, *P* = 0.887). The subjective sensory rating of Dryness only saw a significant difference found between Fabrics B and C (post hoc test, *P* = 0.01).

**Table 5:** Subjective sensation evaluated by questionnaire in response to fabric stimuli.

Sensation	Mean (95% Confidence Interval)			One-way ANOVA	
	Fabric A	Fabric B	Fabric C	*P*-value	*F*-value
Warmness	3.5(3.0–4.0)	2.5(1.8–3.2)	4.9(4.0–5.7)	0.000**	13.0
Dryness	5.4(4.8–6.1)	4.7(3.9–5.6)	5.9(5.4–6.5)	0.04*	3.6
Non-itchy	6.3(5.8–6.8)	6.8(6.5–7.1)	3.7(2.7–4.7)	0.000**	29.6
Non-scratchy	6.6(6.1–7.2)	6.9(6.8–7.1)	3.9(2.9–4.8)	0.000**	31.5
Non-prickly	6.6(6.1–7.2)	6.9(6.8–7.1)	4.5(3.4–5.6)	0.000**	15.6
Smoothness	5.9(4.0–5.8)	6.6(6.2–6.9)	2.9(2.1–3.8)	0.000**	28.7
Non-adhesive	5.1(4.3–6.0)	6.6(6.3–7.0)	3.5(2.6–4.4)	0.000**	20.9
Pliableness	5.4(4.7–6.2)	6.8(6.5–7.0)	4.2(3.4–5.0)	0.000**	17.3
Thickness	5.3(4.4–6.1)	6.3(5.5–7.1)	2.7(2.1–3.4)	0.000**	26.3
Softness	5.4(4.6–6.2)	6.4(6.0–6.9)	3.9(3.1–4.8)	0.000**	14.0
Elasticity	3.7(2.9–4.5)	5.9(5.2–6.5)	3.6(2.8–4.5)	0.000**	12.8
Fullness	3.1(2.1–4.2)	4.3(3.1–5.5)	3.9(3.0–4.9)	0.26	1.4
Comfort	5.4(4.7–6.1)	6.6(6.1–7.1)	4.0(3.2–4.8)	0.000**	17.8

**P <0.01

* *P* <0.05.

### Relationship between EEG relative power, subjective sensations and fabric physical properties

Table 6 illustrates the connection between the subjective sensations of 12 questionnaire participants and the EEG relative power in the Theta and Gamma bands of 21 channels. The power in the Theta band was significantly correlated with all subjective sensory properties (*P*<0.05), except Dryness (*P* = 0.47) and Thickness (*P* = 0.12). The power in the Gamma band was significantly correlated with all subjective sensory properties (*P*<0.05), except Warmness (*P* = 0.16), Non-scratchy (*P* = 0.07), Non-adhesive (*P* = 0.16) and Thickness (*P* = 0.20). Both the powers in the Theta and Gamma bands were significantly correlated with Overall Comfort (*P*<0.05).

**Table 6:** Correlation of the Theta and Gamma bands with the subjective sensations measured by questionnaire.

Band		Warm-ness	Dry-ness	Non-itchy	Non-scratchy	Non-prickle	Smooth-ness	Non-adhesive	Pliable-ness	Thick-ness	Soft-ness	Elasticity	Full-ness	Comfort
Theta	Correlation coefficient	.09*	.004	.23**	.17**	.19**	.21**	.16**	.20**	.05	.24**	.17**	.13**	.34**
	*P* value	.02	.47	.00	.00	.00	.00	.00	.00	.12	.00	.00	.00	.00
Gamma	Correlation coefficient	.04	.20**	-.10*	-.06	-.10*	-.15**	-.04	-.24**	-.03	-.13**	-.20**	-.13**	-.063*
	*P* value	.16	.00	.01	.07	.00	.00	.16	.00	.20	.00	.00	.00	.05

**P <0.01

* *P* <0.05; Theta: θ; Gamma: γ.

Table 7 illustrates the correlations between the EEG relative power in the Theta and Gamma bands of 21 channels and the physical qualities determined by FTT. Results revealed a significant correlation between the relative power in the Theta band and the physical properties of CRR (*P* = 0.000), SFC (*P* = 0.05), and SRW (*P* = 0.009). Significant negative correlations were found between the EEG relative power in the Gamma band and the compression-related properties, CW (*P* = 0.006) and CRR (*P* = 0.000); the bending properties, BAR (*P* = 0.006); and the surface properties, SFC, SRA, and SRW (all *P<*0.05). A significant positive correlation was observed between the EEG relative power in the Gamma band and Qmax (*P* = 0.01).

**Table 7:** Correlation of the Theta and Gamma bands with the physical properties.

		Compression		Thermal	Bending		Surface		
Band		CW	CRR	Qmax	BAR	BW	SFC	SRA	SRW
Theta	Correlation coefficient	.070	.144**	-.061	.070	.046	.076*	.064	.101*
	*P* value	.071	.000	.113	.06	.268	.05	.124	.009
Gamma	Correlation coefficient	-.107**	-.173**	.098*	-.108**	-.076	-.119**	-.102*	-.148**
	*P* value	.006	.000	.011	.006	0.067	.004	.014	.000

**P <0.01

* *P* <0.05; Theta: θ; Gamma: γ.

## Discussion

The research purpose of this study was to use EEG spectral intensity to investigate the tactile sensory response to different textile fabric stimulations. The obtained results suggest that Theta and Gamma power intensities could successfully discriminate the fabric differences in the transient EEG responses and demonstrate high discriminative resolution. The EEG frequency bands also demonstrated a significant correlation with the subjective sensations evaluated by questionnaire and fabric physical properties. This indicated the feasibility of using EEG relative spectral power as a measure to reveal the cortical sensory responses to fine tactile stimuli, which has not been reported before.

The textile industry’s traditional approach involves direct inquiry by questionnaire to ascertain the sensation towards fabric’s tactile stimuli with different textiles. This study saw three different fabric samples in common usage evaluated by questionnaire (Fig 5), with the results indicating that respondents were only able to tell the differences between Fabric C and fabrics A and B on the majority sensations. However, the differences between Fabrics A and B were not recognized in the questionnaires’ analysis. Interestingly, similar results between Fabric C and the other two fabrics were obtained from the EEG relative power, mainly in the Theta and Gamma bands, as shown in Fig 4. Here, the Theta power increased during the static fabric stimuli, while the power of the Gamma wave decreased, compared with the baseline status. Increased Theta and Gamma variations in response to touch have been reported by Michail et al. [25] when comparing the sensory responses caused by tactile and nociceptive stimuli (i.e., pain). High amplitudes of Theta oscillations have been shown to reflect the involuntary attention drawn by novel and salient sensory stimuli [2, 25]. Furthermore, the amplitude of the Theta oscillation was positively associated with the intensity of stimulation [2]. According to Michail et al., only two stimuli were adopted, i.e., either touch or pain stimuli [25]. It was discovered that the Theta power was sensitive enough to identify the stimuli differences by touch (i.e., static loading) in the fabrics. In the case of Gamma wave oscillation, it was found to be positively related to pain stimulation, yet negatively associated with touch stimulation [25], while attended nociceptive stimuli exhibit an increase in neuronal gamma activity [26]. In this study, the relative power of the Gamma waves was reduced in response to all fabric stimulation. This suggests that none of them generated nociceptive sensation in the test. Furthermore, the extent of the Gamma power reduction was also sensitive enough to determine the different fabric stimuli with statistical significance. Gamma oscillations are related to perception, stimulus specificity, and higher-level cognition [27]. In the present study, Fabric C evoked intense tactile stimuli in the form of a scratchy and prickly sensation, which consequently induce uncomfortable perception. It explains the relatively stronger responses of Theta and Gamma waves when touching Fabric C as compared with the other two samples. The responses of waves also suggest they could be used for the perceptive differentiation of fabric tactile stimuli by static loading. The cortical locations of the significant changes in the Theta band were mainly in the left and central positions of the primary somatosensory area, due to the unilateral stimuli on the right forearm (Fig 3). The bilateral distribution of the Gamma variations may be due to the association process in sensory cognition [25]. The findings of the cortical location to stimuli may help researchers in further study on the mapping of neural sources.

As shown in Table 6, most of the subjective sensation properties in the questionnaire showed a significant correlation with Theta and Gamma bands. For instance, the former was found to be significantly related to the sensations of Warmness, Non-itchy, Non-prickle, Non-scratchy, Smoothness, Non-adhesive, Softness, Pliableness, Elasticity, Fullness, and overall Comfort. These sensations address different aspects of the neurophysiological mechanism of touch perceptions [1, 7]. Warmness (Cool-Warm) reflected thermal stimuli and its formation occurred during an extremely short period in contact with the fabric. Fullness, Elasticity and Pliableness related to proprioceptive stimuli, while the tactile sensations of Itchy, Scratchy, Prickle, Smoothness and Non-adhesive referred to an overall description of both irritants and cutaneous stimuli [1, 7]. The sensation of Comfort (Uncomfortable-Comfortable) is an overall indicator of touch perception to the fabric. It is a state of multiple interactions with the surrounding environment and involves physical, psychological, and physiological factors [28]. This may suggest that Theta power changes could be utilized to detect the initial stimuli and directly-perceived sensations felt by the subjects when the fabrics’ stimuli were transferred into nervous signals and broadcast in the brain’s somatosensory cortex. The Theta power could also reflect the perception of overall comfort which was a combined sensation when touching the textile materials and feeling the surrounding environments. Meanwhile, the Gamma power has relationships with sensations of Dryness, Non-itchy, Non-prickle, Smoothness, Softness, Pliableness, Elasticity, Fullness, Fullness and overall Comfort, which reflects the moisture proprioceptive and overall stimuli. However, there are less direct cutaneous stimuli like Scratchy and Adhesive compared to the Theta band. It has been established that Gamma bands are highly associated with cognition from a previous study [27]. Therefore, it can be assumed that the cognitive processes of sensation occur during stimulation of the fabric and could be addressed by the Gamma band. Rating the fabric, via questionnaire, during stimuli was a cognitive behavior. This may further explain the relationship between the responses on Gamma bands and subjective sensation evaluated by questionnaire.

The relationship of the EEG frequency bands and the physical properties of fabrics tested by FTT (Table 7) revealed a significant correlation between the Gamma band and all physical parameters, except for the BW. The Theta band indicated a significant correlation with CRR, SFC, and SRW. Moreover, the relatively low correlation coefficients observed might have been due to the limited values of the fabrics’ physical properties, making it difficult to draw a conclusion regarding the linear relationship between EEG frequency bands and the properties themselves. However, the physical properties of fabrics, such as thermal conductivity, fabric shear, tensile, bending and formability properties have been found to have a relationship with human subjective sensations and Overall Comfort in several studies [5, 7]. As Liao et al. [1, 7] suggested, the sensations of Fullness, Warmness, and Dryness were all under the interacted effects of the fabric properties of the compression module (CW and CRR), the thermal module (Qmax) and the surface module (SFC, SRA and SRW) from the FTT testing; the sensations of Itchy, Scratchy, Prickly, Adhesive and Smoothness were all related to the physical properties on the surface modules (SFC, SRA and SRW); the Pliableness, Elasticity and Fullness sensations correlated to the Bending module (BAR) [1, 7]. Therefore, based on the relationship between subjective sensations and fabric physical properties, it can be speculated that the interacted effects of the fabric surface stimulate different EEG relative power, especially in the Theta and Gamma bands. Converging evidence suggests that the EEG relative power bands could feasibly be used to determine the stimuli from the skin and collect perceptions in the brain. In principle, if more data had been collected from various fabrics and different participants, the transferring model between the fabric physical properties, subjective sensations and EEG power bands could be established, aiding further investigations in the future.

## Conclusions

In this study, EEG spectral analysis was used to investigate the fabric tactile stimulation with different textile compositions, in which 12 young healthy adults (age = 24.1 ± 3.4yrs, 4 males and 8 females) were recruited. Meaningful results with statistical significance have been observed. EEG frequency spectral analysis demonstrated discriminative resolution to fabric stimuli with varying textile compositions. Specifically, the Theta and Gamma powers indicated significant variations in response to the unilateral fabric stimuli to the forearm. The EEG frequency bands also demonstrated a significant correlation with the subjective sensations evaluated by questionnaire and fabric physical properties tested by FTT. Therefore, EEG relative spectral power could be a feasible method of objectively and quantitatively demonstrating sensory perceptions during stimulation. This finding may provide evidence for further exploratory research of sensory perceptions by EEG spectral analysis. It could also be applied to the study of brain generators of skin tactility and the automatic detection of sensory perception in industries.

## Limitations

The main limitation of this study is the small sample sizes of participants and fabrics. Despite the relatively small number of participants recruited, meaningful conclusions were observed. Cross-comparisons on age and gender effects on the variation of EEG responses to textile fabric stimulation with larger sample sizes will be conducted in our future studies. Further investigations on the detailed cortical locations in the sensorimotor cortex and neural connections with more advanced EEG and fMRI cross-measures will be also performed out in the future.

## List of abbreviations

EEG: Electroencephalography
FTT: Fabric Touch Tester
EMG: Electromyography
fMRI: functional magnetic resonance imaging
ERP: Event-related potential
SD: Standard deviation
MMSE: Mini-Mental State Examination
δ: Delta band
θ: Theta band
α: Alpha band
β: Beta band
γ: Gamma band
AATCC: American Association of Textile Chemists and Colorists
ANOVA: Analysis of Variance
CW: Compression Work (gf*mm)
CRR: Compression Recovery Rate (Non-unit)
CAR: Compression Average Rigidity (gf*mm^-3^)
RAR: Recovery Average Rigidity (gf*mm^-3^)
TCC: Thermal Conductivity when Compression (W*m^-1^*K^−1^)
Qmax: Maximum Thermal Flux (W*m^-2^)
TCR: Thermal Conductivity when Recovery (W*m^-1^*K^−1^)
BAR: Bending Average Rigidity (gf*mm*rad^-1^)
BW: Bending Work (gf*mm*rad)
SFC: Surface Friction Coefficient (Non-unit)
SRA: Surface Roughness Amplitude (μm)
SRW: Surface Roughness Wavelength (mm)
